# Effect of perceived interpersonal closeness on the joint Simon effect in adolescents and adults

**DOI:** 10.1038/s41598-020-74859-3

**Published:** 2020-10-22

**Authors:** Raheleh Shafaei, Zahra Bahmani, Bahador Bahrami, Maryam Vaziri-Pashkam

**Affiliations:** 1 School of Cognitive Sciences, Institute for Research in Fundamental Sciences (IPM), Opposite the ARAJ, Artesh Highway, P.O. Box 19395-5746, Tehran, Iran; 2 Department of Biomedical Engineering, Tarbiat Modares University (TMU), Tehran, Iran; 3 Faculty of Psychology and Educational Sciences, Ludwig Maximilian University (LMU), Munich, Germany; 4 Department of Psychology, Royal Holloway University of London, London, UK; 5 Center for Adaptive Rationality, Max Planck Institute for Human Development, Berlin, Germany; 6 Laboratory of Brain and Cognition, National Institute of Mental Health, Bethesda, USA

**Keywords:** Psychology, Human behaviour, Neuroscience, Cognitive neuroscience, Development of the nervous system, Emotion, Social behaviour, Social neuroscience

## Abstract

Here, we explored the role of perceived interpersonal closeness in joint action using the joint Simon task in adolescents and adults. In a two-choice reaction time task, spatially assigned responses to non-spatial stimulus features are faster when the stimulus and response are in congruent locations than not. This phenomenon is called Simon effect and is absent or strongly attenuated when a participant responds to only one of the stimuli. However, the effect reappears when two participants carry out the same go/no-go tasks cooperatively. This re-emergence of the Simon effect in joint action is called the joint Simon effect (JSE). In this study, we first replicated the standard and joint Simon effects in adolescents (n = 43), as well as adults (n = 39) with similar magnitude of the effects in the two age groups. The magnitude of the JSE was positively correlated with the level of closeness as measured by Inclusion of Other in the Self scale. This correlation was not significantly different in adolescents (n = 73) compared to adults (n = 71). Our findings show that joint action is sensitive to the social factor such as interpersonal closeness, and the underlying mechanisms are already mature by adolescence.

## Introduction

In many social interactions, one’s performance is affected by others’ actions, often unwittingly^1^, even when attention to or coordination with the other person is not required. This phenomenon manifests in many non-verbal interactions in everyday social life such as dancing, joint object manipulation, group exercises, group games, or competitive sport. For example, it has been shown that pairs of players who perform an independent competitive task, imitate each other unwittingly such that the performances of the individuals in the dyads are correlated^2^. Or people behave more cautiously (more slowly and accurately) when they observe another person successfully inhibit an action or make an error^3^. Understanding the underlying mechanisms and factors that influence such effects in joint action is essential, given the pervasiveness of joint activities in everyday human life.

The social variant of the Simon spatial-compatibility task^4–6^, termed joint Simon task^7^, has provided a common and powerful laboratory tool to study joint action. The standard Simon task is a two-choice reaction time (RT) task in which single participants give spatially specified responses to non-spatial stimulus features regardless of which location they appear on the screen. Responses are faster and often more accurate when stimulus and response locations are congruent than when they are incongruent^4–6^. For example, subjects are asked to respond to a green or a red stimulus with their left or right hand, respectively. The stimuli can appear on either side of the screen. When the stimuli appear on the side compatible with the responding hand (left for the green or right for the red stimulus), the responses are faster than when they appear on the opposite side. This effect, called the Simon effect, is quite robust and is not affected by variations in stimulus type, response modality, or hand posture^5,8,9^. Simon effect usually disappears when a subject responds to only one of the stimuli, ignoring the other so that the task becomes a simple go/no-go task^7,10^. Interestingly, the effect emerges again in the joint Simon task, where the two complementary go/no-go tasks are carried out by two separate individuals sitting alongside each other. This phenomenon is named the Joint Simon Effect (JSE). In the joint Simon task, each actor is responsible only for a part of the standard Simon task according to the predefined individual rules. But each actor is influenced by the responses of the other actor and performs the shared task similar to performing the whole task alone.

The underlying mechanisms and the social nature of the JSE are still debated (see^11^ for the review). One account of the JSE, called the action co-representation account^12,13^, suggests that during the joint Simon task, participants represent not only their own share of the task but also their partners’ as if it is their own. They integrate their co-actors’ action options into their own action planning^14–16^. This account of the JSE is derived from the ideomotor, or common coding theory of motor programming, which posits that perceiving others’ actions, automatically activates the same motor programs that are involved when one performs those action^17,18^. The action co-representation account of the JSE assumes a link between perception and action during social interactions. Another account of the JSE, referential coding^19^, relies on the fact that the presence of dynamic events that attract attention (e.g., a waving cat, a clock with a rotating element, or a ticking metronome) in place of the co-actor, can induce the JSE^19^. This account suggests that any such sufficiently salient event provides a spatial reference relative to which the participants’ actions will be coded. This referential spatial code will then be either compatible or incompatible with the position of the target and induces the JSE^19,20^. The two accounts of the JSE differ in their predictions about the social nature of the phenomenon. Proponents of the action co-representation account emphasize the social nature of the phenomenon^7,21–23^, while the proponents of the referential coding account argue that any salient event, regardless of its social nature, could induce the JSE^19,20^.

In this study, to contribute to the debate over the social nature of the JSE, we examined whether the perceived interpersonal closeness in the relationship between actors has a role in joint action. Specifically, we asked if the JSE is correlated with the perceived interpersonal closeness between the members of a dyad or not. If the JSE is a social phenomenon, we should be able to observe a correlation with social factors. In line with this hypothesis, some studies have shown that the JSE varies according to interpersonal social factors. The magnitude of the JSE is positively correlated with the passion felt for the romantic partners^24^, or empathy among friends^25^. Furthermore, the magnitude of the JSE is larger when participants co-acted with their romantic partners than when they did with their friends of opposite sex^24^. Also, the JSE is observed when the co-actor is friendly and cooperative as opposed to intimidating and competitive^26^, or is from a perceived in-group member as opposed to an out-group member^27,28^. It is similarly more pronounced among people who have interdependent rather than independent self-construal^29^. Moreover, intake of oxytocin, a neuropeptide that boosts various pro-social behaviors in humans enhances the JSE^30^. These results suggest that the nature of the relationship between the pair should be associated with the magnitude of the JSE. Despite these advances, a study that addresses the effect of real (rather than induced) interpersonal relationship on the JSE, that would include various types of relationships, is missing in the literature. The aim of this study is to measure the effect of interpersonal closeness on the JSE across development from adolescence to adulthood.

The JSE has been previously observed in 4–5-year-old children^31,32^. This observation suggests that the cognitive characteristics that lead to the JSE should be already present in adolescents, and so the JSE should be detectable in adolescence as well. However, it is unknown whether the magnitude of the JSE and how it is associated with the interpersonal closeness of the participants would be the same between adolescents and adults. During adolescence, social and emotional skills, such as self-awareness and understanding of others and their underlying neural mechanisms undergo substantial developments^33–37^. These differences are mainly due to changes in hormone levels as well as structural and the functional changes in certain regions of the social brain^33^, especially the prefrontal cortex (PFC), which is involved in the executive and regulatory functions such as decision-making and affective control^34^. These neural and behavioral variations could impact the magnitude of the JSE and the degree to which it is influenced by the closeness of the participants. On the one hand, an undeveloped understanding of others, and thus weaker action co-representation in adolescents relative to adults might reduce the magnitude of the JSE. On the other hand, greater peer conformity in this age group might enhance it.

Here, we implemented the joint Simon task in two groups of participants: adolescents and adults. We used the Inclusion of the other in the self (IOS) scale to specify the degree of closeness the individuals in a pair felt towards each other^38^. The IOS scale is a simple single-item and user-friendly pictorial tool for measuring the subjectively perceived closeness of a relationship^38^. It is a reliable measure that is highly correlated with other conceptually different, yet more complex and comprehensive, measures of subjective closeness^38,39^. This scale has been extensively used in previous studies to measure the level of closeness between individuals^39^. It has been recently verified as a tool for measuring non-close relationships as well as the close ones^39^ and is intended to cover both conscious and unconscious sense of closeness^38^. We explored the relationship between the IOS measure and the JSE and compared it across the two age groups.

## Method

### Participants

A total of 154 individuals (77 pairs) consisting of 81 adolescents (73 females, age range 13–18 years, *M* = 15.65, SD = 1.28) and 73 adults (43 females, age range 19–37 years, *M* = 26.74, SD = 4.12), participated in the study. We used correlation coefficient of 0.3 as an a-priori measure of effect size derived from previous studies that have looked at the relationship between the magnitude of the JSE and interpersonal affective measures^24,25^. Based on this expected effect size, to have a power greater than 0.8, a sample size of at least 67 participants was required in each of the age groups.

We did not confine our sampling to special types of relationships, and recruited, by advertisement, individual participants or pairs of participants that had relationships including couples, family members, friends, boyfriends/girlfriends, classmate or colleagues. People who were recruited as individuals were matched randomly to other individuals. The number of recruited people and where they came from was as follows: 44 adolescents from school A, 14 adolescents from school B, ~ 20 adolescents from the study hall of a library, ~ 20 adults from students of one department in university A, and ~ 56 adults from researchers of one department in institute B and their connections. The individuals in a pair were either both adolescents or both adults (not necessarily from the same sex) except for one pair in which one individual was an adult (19 years old) and one an adolescent (17 years old). Participants all had normal or corrected-to-normal vision except for one adult male who was removed from the study due to low visual acuity. All participants were right-handed, except for six adolescents and eight adults that were left-handed, and one adolescent that was ambidextrous.

Participation was voluntary, and all participants were naïve to the purpose of the study. Informed consent was obtained from all adults and the parents or school administrators of all adolescents included in the study. All methods were carried out in accordance with relevant guidelines and regulations of the ethics committee at the Institute for Research in Fundamental Sciences (IPM), and SCS Research Ethics Committee of IPM approved the experiment.

### Apparatus and stimuli

The experiments were run using Psychophysics Toolbox in MATLAB 2010b, on PC laptops with a resolution of 1366 × 768, 60 Hz. A small dark circle (0.3°) was presented as a fixation point at the center of the computer screen during an experimental block. Stimuli were red and green circles with a visual angle of 2.86°. In each trial, one of the two stimuli appeared 4.3° either to the left or to the right side of the fixation point. The fixation point was black in between the trials and turned into dark blue during stimulus presentation. Participants sat approximately 60 cm away from the screen during the experiment.

### Procedure

The experiment consisted of three main tasks: (a) individual two-choice (I2C) task i.e., the standard Simon task, (b) individual Go/No-Go (IND) task, and (c) joint Go/No-Go (JNT) task i.e., the joint Simon task.

**Figure 1:**
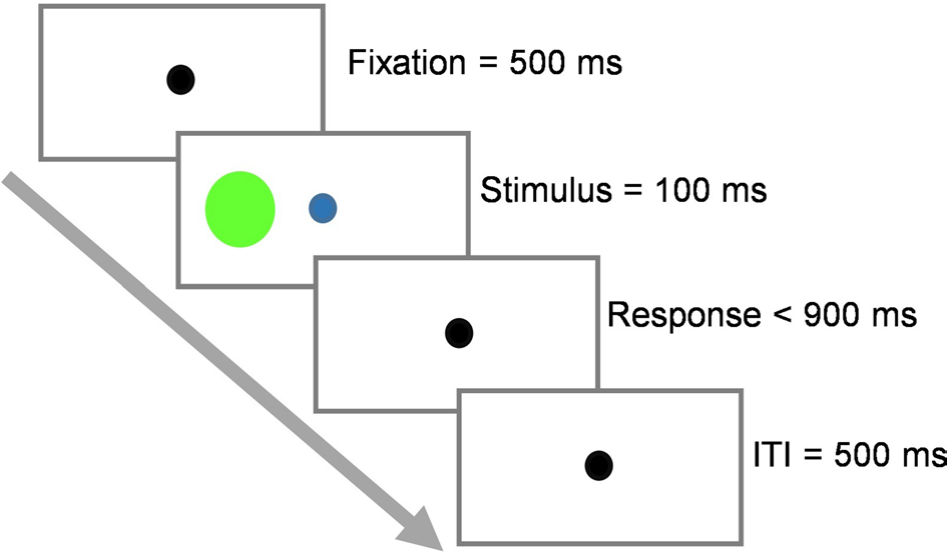
The schematic of a trial. Each trial began with the presentation of the fixation point for 500 ms. The target stimulus was then presented for at most 100 ms. Subjects had 1000 ms to respond following stimulus onset. An inter-trial interval (ITI) of 500 ms, following the response execution or the time-out, concluded the trial.

In the I2C tasks, each individual was instructed to use the left and right keys to discriminate the color of the circles (ignoring the location). Using a computer Keyboard, they pressed the “left shift” key for the red target and the “right shift” key for the green target with the index fingers of their left and right hands, respectively. After the I2C task was completed, the individuals in a pair were randomly assigned one of two colors (red or green) to perform the IND and then the JNT tasks. In both the IND and the JNT tasks, participants only responded to the stimulus with the color they were assigned to with the index fingers of their preferred hands, ignoring the other stimulus. In the congruent trials, the red circle, to which participants responded with the left shift key, appeared on the left side of the screen (congruent with the position of the key), or the green circle, to which participants responded with the right shift key, appeared on the right side of the screen. In the incongruent trials, the positions were reversed. Participants were asked to fixate on the fixation point at the center of the screen and respond as quickly and as accurately as possible. In all tasks, each trial (illustrated in Fig. 1) began with a black fixation point presented for 500 ms. The target stimulus then appeared on the screen for 100 ms, and the fixation point turned blue. After 100 ms, the stimulus was removed and only a black fixation point remained on display. The stimulus color and position were counterbalanced across trials in each experimental block. The trial ended when subjects responded at any time after the onset of the stimulus. The participants were required to respond within the 1000 ms following the onset of the stimulus. If they failed to respond within this time window, the trial would be terminated and recorded as a missed trial. Reaction times (RTs) were measured as the time between the onset of the stimulus and the response. A 500 ms blank screen with a black fixation point in the middle separated the trials.

Participants performed the I2C task and the following IND task on their own, sitting centered in front of the monitor while they were alone in the room. The pair performed the JNT task together while they sat side by side in front of the monitor. The I2C task consisted of 20 training trials, where people received feedback for their responses and four blocks of 80 test trials without feedback. The IND and JGNJ tasks consisted of four blocks of 120 trials without feedback. People were allowed to take short rests in between blocks and tasks.

Eighty-six participants, including 46 adolescents (45 females) and 40 adults (24 females), completed all the three I2C, IND and JNT tasks in a single session that lasted almost an hour. The data from these participants were used to check for and compare the congruency effect across the three tasks in adolescents and adults. In order to collect enough data to explore the association between the JSE and perceived closeness, another 68 participants, including 35 adolescents (28 females) and 33 adults (19 females) were recruited. To make the study more feasible, the new participants performed only the JNT task in a shorter session that lasted ~ 20 min. The data from these latter participants together with the JNT data of those who performed all the three tasks were included in the analysis of the mentioned association. After completing the task(s), participants were asked to fill out a questionnaire about their relationship status individually without sharing their answers.

### Measuring interpersonal closeness

**Figure 2:**
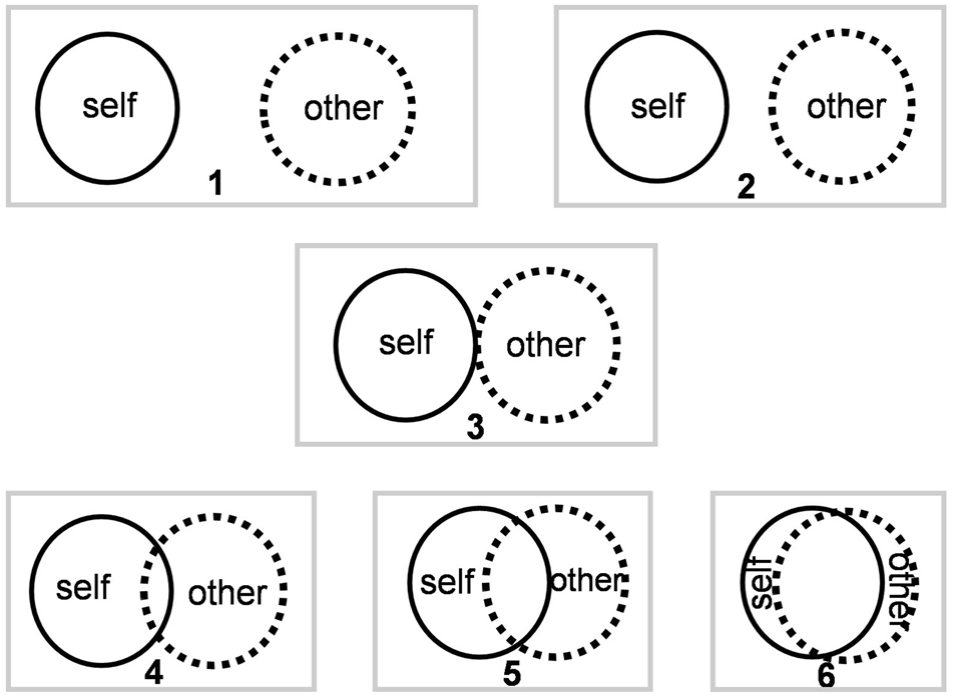
The modified version of the IOS scale used in the study. The solid circles represent the ‘self’ and the dotted circles represent the ‘other’. The cases with no overlap between circles represent negative closeness (i.e., distance or animosity). Closer circles indicate a closer relationship. Therefore, the pairs enclosed in boxes correspond to: 1-moderately distant, 2-slightly distant, 3-neutral, 4-slightly close, 5-moderately close and 6-extremely close perceived feelings toward the other.

After finishing the main tasks, via a questionnaire in Persian language, participants evaluated the degree of familiarity with their experimental partners to be one of four alternatives: stranger, slightly acquainted, quite familiar, and in a close relationship (see Supplementary Appendix S1 for more details). They then were asked to fill out a questionnaire containing the Inclusion of Other in the Self (IOS) scale to rate the degree of closeness they felt towards their experimental partners. In its standard form, the IOS scale consists of seven Venn diagram-like pairs of circles. The individual circles in a pair represent either the “self” or the “other” and vary in their amount of overlap. This overlap is an indicator of the degree of interconnectedness, with higher overlaps indicating closer relationships. We modified the IOS scale originally reported by Aron et al.^38^ in order to include the sense of interpersonal distance, in addition to closeness, by moving the circles away from each other. As such, we had six Venn diagram-like pairs of circles, as depicted in Fig. 2, to measure the level of closeness. The pairs 1–6 corresponded to *moderately distant, slightly distant, neutral, slightly close, moderately close and extremely close* feelings towards the other individual in the pair, respectively. Participants were asked to select the depiction that best described their sense of interconnectedness at the time of the experiment.

### Analysis

We first focused on RTs in the subset of participants who performed all three tasks to analyze congruency (Simon) effects in the I2C, IND, and JNT tasks in our two age groups. Missed trials, incorrect trials, and trials with RTs outside the range of median RT ± 3 × median absolute deviation (MAD, Leys, Ley, Klein, Bernard, & Licata, 2013), were removed from the analysis (on average 24.04, 6.68, 7.44 percentage trials were removed from the I2C, IND and JNT tasks respectively). Four participants (three adolescent females and one adult male) were excluded from further analysis as more than 30% of their trials were removed in at least one of the tasks. In sum, 43 adolescents and 39 adults were included in the analysis.

The analysis of congruency effects across tasks and age groups was performed using ANOVA with a Greenhouse–Geisser correction where necessary. To check for the Simon (congruency) effect, we compared the RTs in the congruent condition to those in the incongruent condition in each of the three tasks (I2C, IND, and JNT) and each of the two age groups. The congruency effect was then calculated as the mean RT on incongruent trials minus the mean RT on congruent trials in any task and for each participant. False Discovery Rate correction^40^ was applied in all cases that multiple comparisons were performed.

We then explored the association between the JSE and perceived interpersonal closeness. We continued collecting data until we had enough samples based on our power analysis, at least 10 participants in the IOS indices 3 to 6 in each age group, and an approximately equal total number of participants across the two age groups. Since only five adolescents reported the sense of distance rather than closeness towards their partners, it was not possible to study the effect of aversive relationships on the JSE. Because we did not have a comparable sample in adults, and because with only 5 samples we would not have a good estimate of the JSE effect in this low-IOS group, we excluded these samples from further analysis and investigated neutral to extremely close interpersonal feelings in our study. Finally, we had 19, 17, 20, and 17 adolescents, and 10, 22, 24, and 15 adults reporting closeness indices of 3 (*neutral*), 4 (*slightly close*), 5 (*moderately close*) and 6 (*extremely close*), respectively, corresponding to the panel numbers in the Venn diagrams of Fig. 2. Following our procedure of data collection there was no significant difference between our adolescent and adult groups in their IOS measure (*X*^*2*^ (3) = 3.89, *p* = 0.2729).

Even though we collected the familiarity measure as well to ascertain that we were not confined to special relationships, note that the focus of this study was the IOS scale as a measure of closeness. The familiarity measure was not deemed appropriate for the purpose of our study and thus not included in the analysis (see the discussion).

## Results

### Simon effects in adolescents and adults

**Figure 3:**
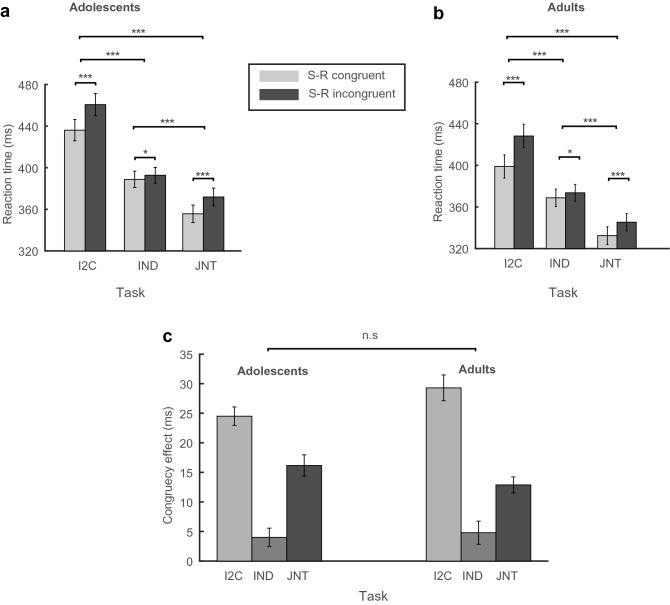
Reaction times and congruency effects for the tasks and the age groups. Top panels: Mean RT for different tasks and spatial stimulus–response (S–R) congruency for (**a**) adolescents and (**b**) adults. Bottom panel (**c**) mean congruency (Simon) effect measured as the RT in incongruent trials minus the RT in congruent trials for different tasks and the two age groups. Error bars indicate standard errors of the means. *p < 0.05, ***p < 0.001.

We first checked for the existence of the congruency effect and its variation across the three tasks of I2C, IND and JNT in adolescents as well as adults in the subset of our participants that performed all three tasks. To establish the presence of the JSE with a high power, it was necessary to compare the behavior of the same participants across the three tasks. This way each participant would be his/her own control. The repeated-measure design applied to tasks makes it possible to remove the variance related to spurious differences between subjects. Accordingly, we ran a three-way ANOVA with age group (adult and adolescent) as a between-subject factor, and congruency (congruent and incongruent) and task (I2C, IND, and JNT) as within-subject factors. This analysis revealed a main effect of congruency (*F*(1,80) = 285.51, *p* < 0.001, ${\eta }_{p}^{2}$ = 0.78), driven by faster RTs in the congruent than incongruent trials; in the I2C and JNT tasks for both adolescents (*ts* > 9.017, corrected *ps* < 0.001, Cohen’s *ds* > 1.37) and adults (*ts* > 9.51, corrected *ps* < 0.001, Cohen’s *ds* > 1.52). The congruency effect was also present in the IND task for both groups (*ts* > 2.45, corrected *ps* < 0.0192, Cohen’s *ds* > 0.39). However, the effect size was much smaller than the I2C and the JNT tasks (note the Cohen’s d values reported above, and see below for more direct comparisons). As depicted in Fig. 3a–c, these results confirmed the existence of standard and joint Simon effects in adolescents as well as adults. The ANOVA also revealed a main effect of age group (*F*(1,80) = 5.02, *p* = 0.028, ${\eta }_{p}^{2}$ = 0.059) with faster RTs in adults than adolescents, and a main effect of task (*F*(1.73,138.13) = 152.36, *p* < 0.001, ${\eta }_{p}^{2}$ = 0.65) caused by faster RTs in the JNT than both the I2C and the IND (*ts* > 11.05, corrected *ps* < 0.001) and in the IND than the I2C task (*t*(163) = 14.37, corrected *p* < 0.001). A significant interaction between congruency and task (*F*(2,160) = 88.98, *p* < 0.001, ${\eta }_{p}^{2}$ = 0.53) was also observed caused by stronger congruency effects in the I2C than both the JNT and the IND (*ts* > 6.67, corrected *ps* < 0.001) and in the JNT than the IND task (*t*(81) = 6.99, corrected *p* < 0.001). There was no significant interaction between congruency and age group (*F*(1,80) = 0.18, *p* = 0.67, ${\eta }_{p}^{2}$ = 0.002), nor between task and age group (*F*(1.73,138.13) = 1.4, *p* = 0.251, ${\eta }_{p}^{2}$ = 0.017). The three-way interaction was not significant (*F*(2,160) = 2.86, *p* = 0.06, ${\eta }_{p}^{2}$ = 0.035).

Even though the three-way interaction was not significant, and there was no indication of an age effect in any of our other analyses, we were curious about the p value approaching significance in the three-way interaction. To further pinpoint where this marginal interaction is coming from, we calculated the congruency effect (Fig. 3c). Post-hoc T tests revealed that there was no significant difference between adolescents and adults in congruency effects across tasks (*ts* < 1.53, *ps* > 0.15). However, the difference between the standard and the joint Simon effects was larger in adults than adolescents (*t*(80) = 2.27, *p* = 0.026). The underlying reason for such a difference is not known to us. Nevertheless, since the JSE was the same across the age groups, this difference was not a critical issue for our further analyses.

There was a high proportion of female participants in our adolescent compared to our adult group. To ascertain, as much as was possible, that this difference did not affect the results, we compared congruency effects across the two genders in adults: there was no gender effect (*F*(1,37) = 0.093, *p* = 0.762, ${\eta }_{p}^{2}$ = 0.003) nor an interaction between gender and task (*F*(1,74) = 0.057, *p* = 0.944, ${\eta }_{p}^{2}$ = 0.002).

Together these results establish the presence of a strong JSE in both age groups and suggest that although adults typically responded faster than adolescents, the magnitude and pattern of congruency effect, and the relative speed across all the tasks are not significantly different between these two age groups.

### Effect of interpersonal closeness on the JSE

Having established the joint Simon effect, we then investigated the relationship between the magnitude of the JSE and the perceived interpersonal closeness. Both participants that completed the three tasks and the ones that only performed the JSE were included in this analysis. To ascertain that this pooling was not problematic, we compared the results of the JSE between these two subsets of participants. Performance (*t*(147) = 1.43, *p* = 0.155), response time (*t*(147) = 1.3, *p* = 0.203) and the magnitude of the JSE (*t*(147) = 1.03, *p* = 0.194) were not significantly different between the two subsets of data.

**Figure 4:**
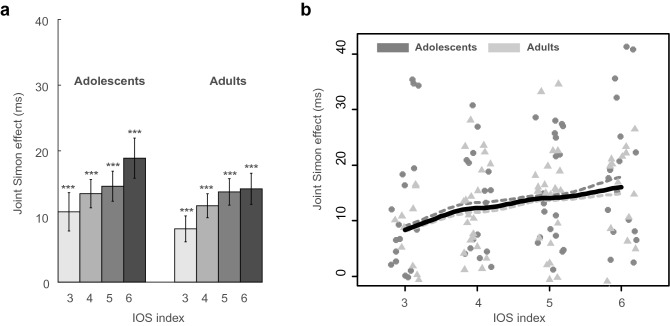
Congruency effects, measured as the RT in incongruent trials minus the RT in congruent trials, for the joint task (JNT) for the two age groups across the IOS scale. (**a**) Mean joint Simon effect for different IOS indices and the two age groups. Error bars indicate standard errors of the means. **p < 0.01, ***p < 0.001 (paired t test, two-tailed). (**b**) Scatter plots of data across IOS indices for adolescents (solid circle) and adults (solid triangle) along with the smoothed lines using the locally weighted smoothing (loess) regression for adolescents (the dashed dark grey line), adults (the dashed light grey line), and both groups together (the solid black line).

As it is illustrated in Fig. 4a, the JSE (measured as the congruency effect in the JNT task) was present in all the IOS indices in both adolescents (*ts* > 3.67, *ps ≤ *0.0018) and adults (*ts* > 4.11, *ps ≤ *0.0026). A Spearman correlation analysis confirmed that the magnitude of the JSE increases with the IOS scale in adolescents (*rs* (71) = 0.261, *p* = 0.026), and the positive correlation is approaching significance in adults (*rs* (69) = 0.22, *p* = 0.067). The difference between these two correlations was not significant (*z* = 0.26, *p* = 0.79). Accordingly, and also relying on the fact that there was no significant difference between adolescents and adults in the JSE nor in the IOS scale, we pooled the results from adolescents and adults together: the JSE across the whole population was positively correlated with the IOS scale (*rs* (142) = 0.23, *p* = 0.006). These results are depicted in Fig. 4b.

Since there could be distinct developmental differences between a 13 and an 18-year-old adolescent, to constrain our age group, and make the claims more robust, we repeated the correlation analysis for adolescents of ages 15–17, which comprised 75% of our adolescents. A significant correlation was still present in this restricted dataset (*rs* (52) = 0.28, *p* = 0.037). The median and standard deviation of the age difference between the individuals in a pair were 1 and 0.68 in adolescents and 3 and 2.87 in adults, respectively.

Again, to check whether the dominance of females in adolescents would be less likely to affect the results, we compared the magnitude of the JSE between the two genders in adults; there was no gender effect (*t*(69) = 0.1, *p* = 0.92).

Also, in order to evaluate whether the obtained measures were affected by our dyadic setting, we measured the correlation coefficient between the magnitude of the JSEs of the two individuals in the pairs. This analysis revealed no significant correlation (*r* (75) = − 0.01, *p* = 0.92) indicating that the magnitude of the JSE of one individual in a pair was not associated with that of their partner.

These results together suggest that the JSE is present for all degrees of subjective closeness from neutral to highly positive in adolescents and adults, and further show that the magnitude of the JSE increases at higher degrees of interpersonal closeness indicating that the quality of the relationship is associated with the way the pairs are influenced by each other’s actions during joint tasks.

## Discussion

In this article, we investigated the effect of interpersonal closeness on the joint Simon effect in adolescents and adults. We first established the presence of the standard and the joint Simon effects in both adolescents and adults. We found no significant difference in the magnitude of the effects between the two age groups. We then showed that there is a significant correlation between the magnitude of the JSE and the level of interpersonal closeness, with higher levels of closeness associated with increased JSE.

Simon effects in adolescents have not been measured before. Even though the mere presence of Simon effects in adolescents could be inferred from previous studies in children^31,32^ and across development (see^34^ for a review), it is not obvious that the magnitude of the effects in adolescents should be the same as that in adults. In addition to demonstrating the presence of both standard and joint Simon effects in adolescents and adults, our results show that the magnitude of these effects is comparable between the two age groups. Previous studies have postulated that the (standard) Simon effect emerges due to a conflict in the response-selection stage of decision-making and a disruption in voluntary response inhibition^10,41,42^. Behavior in paradigms that involve response inhibition, such as go/no-go, Stroop, stop-signal reaction time, and anti-saccade tasks, have been previously compared in children, adolescents, and adults (see^34^ for a review). It has been suggested that the accuracy in response inhibition improves from childhood to adolescence and remains constant from late adolescence to adulthood^43,44^. Our results are consistent with these findings and confirm that adolescents are already mature in cognitive characteristics leading to the standard Simon effect. Furthermore, the presence of the JSE in adolescents with a comparable magnitude as that in adults suggests that the underlying mechanisms that lead to the JSE develop before adolescence and during childhood. This result is in line with and extends the previous findings that have shown the existence of the JSE in children^31,32^.

Although the congruency effects were the same for both groups, in our experiment, adults were typically faster in all tasks. This observation is in line with a large body of literature on the development of processing speed^44–50^, which has shown that on a wide range of speeded motor, perceptual, and cognitive tasks, adults respond faster than (young) adolescents. The underlying mechanism has been suggested to be global and non-task specific but instead, to be a fundamental characteristic of the developing information-processing system.

One limitation of our study is that the participants in our adolescents were dominantly females. Congruency effects were not affected by gender in adults. Given this, it is unlikely that congruency effects would be affected by gender in adolescents. However, this possibility cannot be completely ruled out. This limitation could be remedied in future studies. Unlike some previous findings^7,17,18,51^, we found a significant congruency effect in the IND task in which subjects were performing the go/no-go task individually. This observation implies that the participants in the IND task had encoded both the relevant “go” and the irrelevant “no go” response. There might be two sources for this effect: first, in our experiment, the IND task was preceded by the I2C task, which required the actual execution of the alternative response. Representation of both responses in the I2C task may have transferred to the subsequent IND task^52–54^. Second, because the response buttons (right and left shift keys) were at the two ends of the keyboard, even though the participants were asked to perform the go/no-go task, they may have spatially encoded the buttons, leading to a small but significant Simon effect^55^. Nevertheless, the congruency effect in the IND task was much weaker than that in the I2C and the JNT tasks consistent with previous findings^52–56^, suggesting that the encoding of the irrelevant stimulus was much weaker in the IND than the JNT task.

Comparing response times among the three tasks revealed that consistent with previous studies^18^, the response times in the I2C and the JNT tasks were longer than the response times for the IND task. These results are possibly related to the Hick’s law^57^ that suggests an increase in reaction time with an increase in the number of choices. Furthermore, the response times in the JNT task were faster than those in the IND task. This difference might be caused by a social facilitation effect and is in line with previous studies showing that the performance in simple tasks improves in the presence of or cooperation with others (see^58^ for a review). To ascertain that this pattern of findings is not attributed to a practice effect (i.e., response times decrease over the course of the experiment), we compared the results of the JSE between those participants who performed all three tasks and those participants who performed only the JNT task. There was no significant difference between the performance, reaction time or the effect magnitude across these two subsets.

Our most prominent finding is the positive correlation of the magnitude of the JSE with the feeling of interconnectedness between the individuals in a pair. These results are consistent with previous studies that have shown that competitiveness or friendliness of a partner could change the magnitude of the JSE^26^, that individuals with interdependent self-construal have larger JSEs^29^, and that in-group pairs have stronger JSEs than out-group pairs^27,28^. Our results extend these findings and show that a simple measure of interpersonal closeness could predict the magnitude of the JSE. Consistent with our findings, more recently, Quintard et al.^24^ showed that the magnitude of the JSE was larger when participants co-acted with their romantic partners than when they did with their friends of opposite sex. Moreover, there was a relation between the magnitude of the JSE and a measure of passion felt for the romantic partners. The authors also looked at the effect of IOS but only within each of the individual groups of romantic partners and friends., and failed to show a correlation between the measured IOS scale and the magnitude of the JSE in romantic partners or friends of the opposite sex. Within each group of romantic partners or friends, the reported IOS is likely to cover a narrow range. This narrow range could make it difficult to observe a significant correlation. In another study^25^, Ford and Aberdein measured the JSE when the partners were friends and compared it to when they were strangers. On the one hand, they found no significant difference in the magnitude of the JSE between the two groups. On the other hand, they reported that only in the friends group there was a significant positive correlation between empathy and the magnitude of the JSE. The former finding is ostensibly inconsistent with our results. However, it is worth noting that the longevity of a relationship does not guarantee high levels of closeness since the relationship duration has been shown not to be highly correlated with more rigorous measures of closeness^38^. In other words, strangers do not necessarily feel neutral to each other, or people who have known each other for some time may not feel interconnected. In spite of this, the latter observation of Ford and Aberdin indicates that friends were influenced by the other person’s action in a way that was not true for strangers. In our study, we did not confine our sampling to special types of relationships, and with an established measure of subjective closeness showed the positive correlation of interpersonal closeness with the magnitude of the JSE.

Our findings, in line with previous work^24,25,30^, provide evidence in support of the sensitivity of the JSE to social factors. These results could be explained by both main accounts of the JSE. The action co-representation account of the JSE suggests that in joint actions, individuals partially represent their partners’ as well as their own action. In this context, our findings could mean that individuals represent more of their co-actors’ actions when they feel more interconnected with them. In other words, the closer and more interconnected individuals feel toward their partners, the more difficult it will be for them to discriminate their own actions from their partners’, leading to larger JSEs. This interpretation conspicuously matches the origins of the IOS scale. The IOS scale was originally derived from the self-expansion model. The self-expansion model states that individuals tend to expand their sense of self in order to increase their ability to accomplish goals (see^59^ for the review). According to this model, individuals often use close relationships to expand themselves by incorporating others in the self; that is, individuals behave as though some or all aspects of their close partners, including their resources, viewpoints, traits, and abilities, belong to their own^38,60^. This cognitive viewpoint of closeness is termed inclusion of the other in the self (IOS) and suggests that closeness is defined by overlapping cognitive structures with another individual^38,61^. It is possible that individuals would also regard others’ actions as their own, and more so in their close relationships. If so, our results could be explained by a combination of the self-expansion model and the action representation model, meaning that the JSE arises from representing other’s actions with a stronger representation of the actions of close partners. This is quite in line with the suggestion that the self-expansion model (i.e., reduction in self-other discrimination) is supported in the bodily level as well as the conceptual level (i.e., traits, interests, attitudes) of self-representation^24^.

On the other hand, our results could also be explained by the referential coding account of the JSE. This account assumes that the presence of any salient event induces the JSE, as the salient event provides a spatial reference that induces a spatial congruency effect. It is possible that the feeling of interconnectedness would cause the co-actors and their actions to be more salient, as close others may attract more attention. This effect, in turn, would lead to a larger JSE with an increased level of closeness. Nevertheless, it is important to note that previous studies have only modified saliency through physical properties of the stimulus or the action dynamics^11,19^ and not through the attributes of the relationship. Therefore, to explain our results, the referential coding account would need to be modified to include social factors as possible variables that can modify saliency. This modified version has been proposed by the recently revised theory of event coding^62^.

Regardless of the underlying mechanism, our findings emphasize the social nature of the joint Simon effect. These results suggest that the affective quality of the relationship between collaborators can influence collaborative activity and that higher-order processes can influence joint actions. These results are especially relevant in situations that require selecting partners for particular purposes and suggest that a simple measure of IOS may be revealing of which pair could have the most optimal degree of action coordination. Future research should address this topic and the feasibility of using the joint action paradigms and the IOS scale for matching collaborating partners.

## Supplementary information

Supplementary Information

## Data Availability

The datasets generated during and/or analysed during the current study are available in the GitHub repository, https://github.com/Rshafaei/JSE/blob/master/data.rar, https://doi.org/10.5281/zenodo.3228987.
