# Supplementary material for: Effect of perceived interpersonal closeness on the joint Simon effect in adolescents and adults
Source: Sci Rep. 2020 Oct 22;10:18107. doi: 10.1038/s41598-020-74859-3 (PMC7582195; doi:10.1038/s41598-020-74859-3)
Supplement: Supplementary file 1 — Supplementary Information [file 41598_2020_74859_MOESM1_ESM.docx]

**Effect of Perceived Interpersonal Closeness**

**on the Joint Simon Effect in Adolescents and Adults**

Raheleh Shafaei^1*^

Zahra Bahmani Dehkordi^2^

Bahador Bahrami^3,4,5^

Maryam Vaziri-Pashkam^6^

1. School of Cognitive Sciences, Institute for Research in Fundamental Sciences (IPM), Tehran, Iran.
2. Department of Biomedical Engineering, Tarbiat Modares University (TMU), Tehran, Iran.
3. Faculty of Psychology and Educational Sciences, Ludwig Maximilian University (LMU), Munich, Germany.
4. Department of Psychology, Royal Holloway University of London, London, UK.
5. Max Planck Institute for Human Development, Center for Adaptive Rationality, Berlin, Germany
6. Laboratory of Brain and Cognition, National Institute of Mental Health, USA.

^*^Correspondence concerning this article should be addressed to Raheleh Shafaei, School of Cognitive Sciences, Institute for Research in Fundamental Sciences (IPM), Opposite the ARAJ, Artesh Highway, P.O. Box 19395-5746, Tehran, Iran. +98 21- 26113274. Email: [rshafaei@ipm.ac.ir](mailto:rshafaei@ipm.ac.ir).

**Appendix**

**IOS Questionnaire**

The Persian translation of the following Questionnaire was used:

In the following figure, there are two circles in each panel. One of the circles labeled “self” represents the participant and the other circle labeled “other” represents his/her partner in the experiment. The overlap between the circles demonstrates the level of interconnectedness between the self and the other (the extent to which individuals include their partners in themselves). Therefore, the tangent circles (case number 3) represent the neutral state of closeness in the relationship. The larger overlaps represent a stronger sense of interconnectedness between the individuals and the larger distances between the two circles represent a stronger sense of aversion between the individuals. During the experiment, each participant will be asked to assign a number from 1 to 6 (corresponding to panels 1 to 6) which best describes his/her relationship with their partners.

self

other

1

self

other

2

3

self

other

4

self

other

5

self

other

6

other

self

**Relationship Questionnaire:**

The Persian translation of the following Questionnaire was used:

Participant’s ID: Sex: Age: Handedness (left/right):

How much do you know your partner?

1. I have never seen him/her before: s/he is a stranger to me.
2. I have already seen and known him/her, but I have had little interaction with him/her, only through limited meetings or conversations.
3. I know him/her and I have significant interaction with him/her.
4. I know him/her very well and I have a familial/friendly/spousal relationship with him/her.

Which item best describes your relationship with your partner at the time of the experiment?
